# Predictive value of pre-delivery serum b-human chorionic gonadotropin, fibrinogen, and homocysteine for pregnancy-induced hypertension

**DOI:** 10.5937/jomb0-52483

**Published:** 2025-03-21

**Authors:** Hani Lin, Hong Chen, Miaomiao Zhuo

**Affiliations:** 1 Ruian Maternity and Child Care Hospital, Department of Clinical Laboratory, Rui'an, China

**Keywords:** pregnancy complications, hypertension, beta-HCG, fibrinogen, homocysteine, lipids, receiver operating characteristic curve, komplikacije u trudnoći, hipertenzija, beta-HCG, fibrinogen, homocistein, lipidi, ROC kriva

## Abstract

**Background:**

We aimed to investigate the relationship between pre-delivery serum b-human chorionic gonado - tropin (b-HCG), fibrinogen (FIB), and homocysteine (HCY) with hypertensive disorder complicating pregnancy (HDCP).

**Methods:**

This was a case-control study. 200 HDCP patients and 150 normal pregnant women were selected as study subjects. Fasting cubital venous blood samples were collected to measure serum triglycerides (TG), total cholesterol (TC), high-density lipoprotein (HDL), and lowdensity lipoprotein (LDL), as well as b-HCG, FIB, and HCY levels. Pearson correlation analysis examined the relationship between b-HCG, FIB, HCY, and HDCP. Receiver operating characteristic (ROC) curve analysis evaluated the predictive value of these indicators for HDCP. Multiple logistic regression analysis identified risk factors (RFs) for HDCP.

**Results:**

Serum TG, TC, HDL, LDL, FIB, b-HCG, and HCY were greatly elevated in the HDCP group versus the control group (CG) (P<0.05). Serum b-HCG, FIB, and HCY showed notable positive correlations with HDCP (r=0.935, 0.547, 0.811; P<0.05), and the areas under the ROC curve (AUC) for predicting HDCP based on serum b-HCG, FIB, HCY, and their combination were 0.743, 0.659, 0.801, and 0.886, respectively (P<0.05). Lipid indicators and serum levels of FIB, b-HCG, and HCY were RFs for HDCP.

**Conclusions:**

Pregnant women with HDCP exhibited markedly elevated serum lipid levels and FIB, b-HCG, and HCY levels before delivery, which can serve as predictive indicators for HDCP.

## Abstract (sr)

**Uvod:**

Naš cilj je bio da istražimo odnos između serumskog b-ljudskog horionskog gonadotropina (b-HCG), fibrinogena (FIB) i homocisteina (HCY) pre porođaja i hipertenzije što dovodi do komplikacija u trudnoći (HDCP).

**Metode:**

Ovo je bila studija tipa slučaj-kontrola. Ispitivanjem je obuhvaćeno 200 pacijentkinja sa hipertenzivnim poremećajem tokom trudnoće (HDCP) i 150 trudnica sa normalnim tokom trudnoće. Uzeti su uzorci venske krvi pre uzimanja hrane iz kubitalne vene kako bi se izmerili nivoi serumskih triglicerida (TG), ukupnog holesterola (TC), lipoproteina visoke gustine (HDL) i lipoproteina niske gustine (LDL), kao i b-HCG, FIB i HCY. Korišćena je Pearsonova korelaciona analiza za ispitivanje odnosa između b-HCG, FIB, HCY i HDCP. Analizom krive operativnih karakteristika primalaca (ROC) došlo se do prediktivne vrednosti ovih pokazatelja za HDCP. Multivarijantna logistička regresiona analiza je identifikovala faktore rizika (RF) za HDCP.

**Rezultati:**

Nivoi serumskih TG, TC, HDL, LDL, FIB, b-HCG i HCY su bili značajno povišeni u grupi sa HDCP u poređenju sa kontrolnom grupom (CG) (P<0,05). Serumski bHCG, FIB i HCY su pokazali značajnu pozitivnu korelaciju sa HDCP (r=0,935, 0,547, 0,811; P<0,05), a površine ispod ROC krive (AUC) za predviđanje HDCP na osnovu serumskih vrednosti b-HCG, FIB, HCY i njihove kombinacije su iznosile 0,743, 0,659, 0,801 i 0,886, redom (P<0,05). Lipidni pokazatelji i serumski nivoi FIB, b-HCG i HCY su identifikovani kao faktori rizika za HDCP.

**Zaključak:**

Trudnice sa HDCP su pokazale izrazito povišene nivoe serumskih lipida, FIB, b-HCG i HCY pre porođaja, {to sve mogu da budu prediktivni pokazatelji za HDCP.

## Introduction

Pregnancy-induced hypertension (hypertensive disorder complicating pregnancy, HDCP) is a common pregnancy complication, with an incidence rate of approximately 7-12% [1]. As HDCP worsens, maternal organ and placental function decline, subsequently affecting fetal growth and development and leading to adverse pregnancy outcomes [2]. HDCP pathogenesis is not fully understood, and effective therapeutic interventions are lacking clinically, underscoring the importance of early prediction of HDCP. 

Under normal physiological conditions, the body maintains a dynamic balance between coagulation and anticoagulation functions, where procoagulant and anticoagulant factors interact to collectively regulate the clotting process [3]. In the late stages of pregnancy, women are in a hypercoagulable state, which is advantageous for preventing excessive bleeding during pregnancy. Fibrinogen (FIB) is a glycoprotein synthesized and secreted by liver cells and is the most abundant coagulation factor in plasma. Studies indicated that adverse pregnancy outcomes such as pregnancy-induced hypertension, placental abruption, and recurrent miscarriage may be associated with changes in coagulation function [4] [5] [6]. In addition, HDCP can lead to endothelial injury and dysfunction in parturients, which is a central component of the pathogenesis of HDCP. Homocysteine (HCY) is closely related to endothelial damage, suggesting its potential involvement in developing HDCP [7]. β-human chorionic gonadotropin (β-HCG) is secreted by syncytiotrophoblast cells of the placenta. It helps to maintain the corpus luteum and reduces maternal lymphocyte activity, thus preventing maternal rejection of the fetus. Numerous studies noted a close relationship between β-HCG and the progression of HDCP [8] [9] [10].

However, there is relatively limited research on the changes in serum β-HCG, FIB, and HCY levels before delivery in HDCP patients and their predictive value for HDCP. Despite the established associations between HDCP and changes in coagulation function, endothelial damage, and β-HCG levels, there is a lack of comprehensive studies investigating the predictive value of these biomarkers for HDCP. Specifically, the relationship between pre-delivery serum β-HCG, FIB, and HCY levels and HDCP has not been thoroughly explored. This study aims to fill this knowledge gap by examining the predictive value of these biomarkers for HDCP, which could lead to the developing of novel diagnostic and therapeutic strategies for this condition. This study is novel in its comprehensive investigation of the relationship between pre-delivery serum β-HCG, FIB, and HCY levels and HDCP.

## Materials and methods

### Case information

This was a case-control study. Two hundred confirmed cases of HDCP diagnosed between January 2021 and December 2023 at Ruian Maternity and Child Care Hospital were enrolled. Additionally, 150 pregnant women with normal pregnancies during the same period were set as the control group (CG). All parturients collected blood samples at the age of 15~21 weeks for relevant examination. The diagnosis of HDCP was based on the criteria outlined in the 2020 guidelines of the Chinese Medical Association Obstetrics and Gynecology Branch, which include systolic blood pressure >140 mmHg and diastolic blood pressure >90 mmHg, accompanied by one of the following conditions: pre-existing hypertension; pregnancy-induced hypertension and pre-eclampsia; preexisting hypertension with proteinuria; or unclassified hypertension. Inclusion criteria: age >18 years; naturally conceived singleton pregnancy; normal fetal intrauterine growth; understanding of the study procedures, voluntary participation, and signing informed consent. Exclusion criteria: significant organ dysfunction affecting the heart, liver, or kidneys; history of bleeding disorders or thrombotic diseases; other types of malignant diseases; pre-existing hypertension or diabetes before pregnancy; history of miscarriage or terminated pregnancy; abnormal thyroid function; history of mental disorders or language impairments; recent use of medications affecting coagulation function. This work obtained approval from the Ruian Maternity and Child Care Hospital Ethics Committee.

We estimated the required sample size to achieve a power of 80% and a confidence level of 95% based on a similar study [11]. Assuming a similar correlation coefficient, we calculated that a total sample size of 350 participants (200 cases and 150 controls) would be required to detect a statistically significant difference in serum β-HCG levels between women with hypertensive disorders of pregnancy and those with normal pregnancies.

### Ethical considerations

This study was conducted using the principles of the Declaration of Helsinki, and approval was obtained from the Ruian Maternity and Child Care Hospital Ethics Committee. Informed consent was obtained from all participants before their enrollment in the study, and all data were anonymized and kept confidential to ensure participant privacy. The ethics committee reviewed and approved the study protocol, ensuring that the participant's rights and welfare were protected throughout the study.

### Serum sample collection

Patients in the HDCP group and CG, between gestational weeks 15-20+6, were instructed to collect venous blood (3 mL) in the morning after fasting via the cubital vein. They used vacuum blood collection tubes containing 0.109M sodium citrate and venous blood (5 mL) using vacuum blood collection tubes with a coagulant. Subsequently, using a TDL-40B centrifuge (Shanghai Anting Scientific Instruments Co., Ltd.), the blood was centrifuged at 3,000 rpm for 15 minutes to separate plasma and serum samples. The supernatant was extracted and stored at -70 °C for further testing.

### Detection of relevant indicators in serum

The levels of activated partial thromboplastin time (APTT), prothrombin time (PT), and fibrinogen (FIB) in serum were determined using the CX810 automated coagulation analyzer (Mindray Bio-Medical Electronics Co., Ltd., Shenzhen). Serum triglyceride (TG), total cholesterol (TC), high density lipoprotein (HDL), low density lipoprotein (LDL) and homocysteine (HCY) were measured by Atellica Solution CH automatic assembly line (Siemens, Germany). The instrument used to detect B-HCG in serum is Wallad AutoDELFIA ® 1235 automatic time-resolved fluorescence immunoassay analyzer (PerkinElmer, Inc, USA). And strictly follow the instructions attached to the kit.

### Statistical analysis

All data were entered into an Excel spreadsheet and analyzed using SPSS 23.0. Categorical data were presented as n (%) and analyzed between groups using non-parametric chi-square tests. Normally distributed continuous data were denoted as mean ± standard deviation and compared via Student's t-tests. ROC curves assessed the predictive value of indicators for HDCP. Pearson correlation analysis evaluated the correlation between each indicator and HDCP. Multiple logistic regression analysis was employed to identify RFs for HDCP. P<0.05 denoted statistical significance.

## Results

A comparison of general characteristics between the CG and HDCP groups in parturients was conducted. The groups showed no significant differences in age (28.9±1.7 vs 29.1±2.3 years), gestational week (35.4±2.2 vs 34.8±2.5 weeks), BMI (22.5±4.1 vs 23.0±3.7 kg/m^2^), or several deliveries (84% vs 85% primiparous). However, significant differences were found in systolic and diastolic blood pressure (122.7±6.8 vs 142.1±8.8 mmHg and 76.5±5.3 vs 95.4±7.4 mmHg, respectively), newborn body mass (3.3±0.6 vs 2.8±0.3 kg), and newborn 1-minute Apgar score (9.5±1.1 vs 8.7±0.9), with p-values of 0.021, 0.017, 0.045, and 0.028, respectively (Table 1).

**Table 1:** General information on postpartum women.

Item	CG	HDCP group	*P*
*n*	150	200	
Age, years old	28.9±1.7	29.1±2.3	0.431
Gestational week, weeks	35.4±2.2	34.8±2.5	0.338
BMI, kg/m^2^	22.5±4.1	23.0±3.7	0.440
Number of deliveries, n (%)			0.382
Primiparous	126 (84.0)	170 (85.0)	
Multiparous	24 (16.0)	30 (15.0)	
Systolic blood pressure (mmHg)	122.7±6.8	142.1±8.8	0.021
Diastolic blood pressure (mmHg)	76.5±5.3	95.4±7.4	0.017
Newborn body mass, kg	3.3±0.6	2.8±0.3	0.045
Newborn 1-minute Apgar score	9.5±1.1	8.7±0.9	0.028

**Figure 1:**
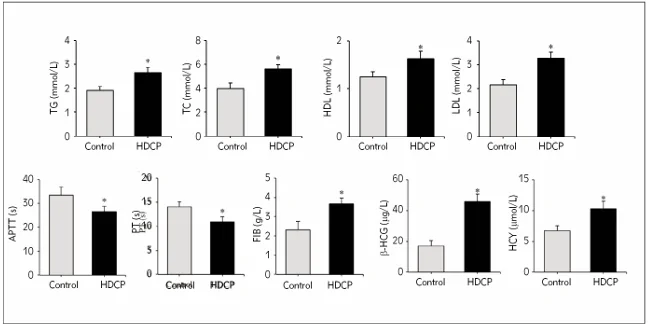
Path diagram of mediation analysis. (Note: Figure 1 represents the mediation analysis path diagram of inflammatory factors on the relationship between glycemic abnormalities and disease severity. A-C represent the mediating effects of IL-6, WBC and hs-CRP, respectively).

A comparison of lipid indicators between the CG and HDCP groups revealed substantial differences. In the HDCP group, serum TG, TC, HDL, and LDL were all considerably superior to CG (P<0.05). Figure 1 illustrates the graphical representation of these findings. A comparison of coagulation function indicators between the CG and HDCP groups was conducted, revealing marked differences. APTT and PT were notably shorter than CG in the HDCP group, while FIB levels were significantly higher than CG (P<0.05). Figure 1 shows the graphical representation of these findings. A comparison of serum β-HCG and HCY levels between the CG and HDCP groups revealed drastically higher levels in the HDCP group versus CG (P<0.05). Figure 1 is the graphical representation of these findings.

**Figure 2:**
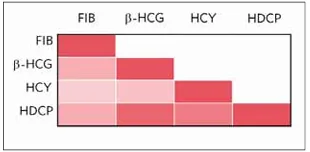
Correlation of serum FIB, β-HCG, HCY, and HDCP.

Pearson correlation analysis revealed that FIB, β-HCG, and HCY serum levels were all positively correlated with HDCP (r=0.547, 0.935, 0.811; P=0.001, 0.000, 0.000). Figure 2 is the graphical representation.

### Predictive value of serum FIB, β-HCG, and HCY levels for HDCP 

**Figure 3:**
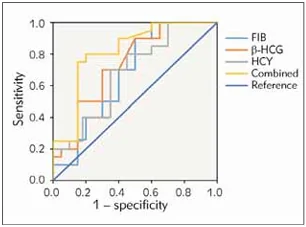
ROC curve of serum FIB, β-HCG, and HCY in predicting HDCP.

ROC analysis assessed the predictive value of serum FIB, β-HCG, HCY levels and their combination in predicting HDCP. The results indicate that the predictive value of single-parameter testing ranks as follows: β-HCG>HCY>FIB. However, the predictive value of the combined three-parameter test surpasses that of individual tests for FIB, β-HCG, and HCY. Figure 3 and Table 2 show the detailed results.

**Table 2:** Predictive efficacy of serum FIB, β-HCG, and HCY for HDCP.

Indicator	AUC	Standard error	*P*	95% confidence interval
FIB	0.659	0.062	0.011	0.610~0.924
β-HCG	0.801	0.070	0.001	0.632~0.950
HCY	0.743	0.066	0.005	0.646~0.948
Three indicators combined	0.886	0.059	0.000	0.765~0.982

A multiple variable logistic regression analysis identified RFs influencing HDCP. The analysis revealed that serum levels of TG, TC, HDL, LDL, FIB, β-HCG, and HCY were all notable RFs for HDCP. Based on odds ratios (OR), the RFs were ranked in order of magnitude as follows: TG>β-HCG>HDL>HCY>FIB>TC>LDL (Table 3).

**Table 3:** Multiple logistic regression analysis of RFs of HDCP.

Indicator	β	Standard error	Wald χ^2^	*P*	*OR*	95% confidence<br>interval
TG	1.541	0.836	2.121	0.015	2.193	1.320~2.874
TC	0.403	0.428	0.566	0.024	1.128	0.718~1.446
HDL	1.282	0.975	1.347	0.033	1.975	1.316~2.413
LDL	0.710	1.143	1.089	0.048	1.054	1.008~2.138
FIB	0.572	0.829	1.954	0.041	1.268	0.954~2.226
β-HCG	0.805	0.551	0.968	0.012	2.035	0.281~0.979
HCY	0.616	0.725	1.143	0.035	1.886	0.589~2.145

## Discussion

This study showed that pregnant women with HDCP exhibited markedly elevated serum lipid levels and FIB, β-HCG, and HCY levels before delivery, which can serve as predictive indicators for HDCP. These results are consistent with previous research on the topic. Yadav et al. [11] also investigated the association between serum β-HCG levels and hypertensive disorders of pregnancy in a prospective observational study involving 200 antenatal women. They found that higher serum β-HCG levels were significantly associated with hypertensive disorders of pregnancy, with high levels correlating with 59 out of 78 cases. In contrast to Yadav et al.'s [11] study, which only examined the relationship between β-HCG levels and HDCP, our study took a more comprehensive approach by examining multiple biomarkers, including serum lipid levels, FIB, and HCY levels. Our findings suggest that these biomarkers and β-HCG levels may serve as predictive indicators for HDCP. Dawle et al. [12] also investigated the relationship between serum β-HCG levels and pregnancy-induced hypertension in a study involving 400 antenatal women. They found that women with high serum β-HCG levels at 12 to 24 weeks of gestation had a 1.67 times higher risk of developing pregnancy-induced hypertension and poor maternal and perinatal outcomes. Consistent with the findings of Murmu and Dwivedi [13], we found that serum β-HCG levels were significantly elevated in the HDCP group compared to the control group (P<0.05). In contrast to Murmu and Dwivedi's study, which focused on the predictive value of serum β-HCG and lipid profile in the second trimester for pregnancy-induced hypertension (PIH), our study examined the predictive value of β-HCG, FIB, and HCY in the pre-delivery period for HDCP. Interestingly, both studies found that lipid indicators were risk factors for hypertensive disorders of pregnancy. Murmu and Dwivedi [13] found that in the early and late second trimesters, serum cholesterol, triglyceride, and very low-density lipoprotein levels significantly increased in PIH patients. Similarly, our study found that serum lipid biomarkers were greatly elevated in the HDCP group versus the control group (P<0.05).

Consistent with the findings of Maru et al. [14], we found that serum HCY levels were significantly elevated in the HDCP group compared to the control group (P<0.05). Maru et al. [14]. also found a significant correlation between serum HCY levels and the severity of hypertension, with higher levels of HCY associated with more severe hypertension (p = 0.759). Our study and Maru et al.'s [14] study both suggest that HCY may be a useful predictive marker for hypertensive disorders of pregnancy. However, our study examined the relationship between HCY and HDCP in the context of other biomarkers, including β-HCG and FIB, whereas Maru et al.'s [14] study focused specifically on the relationship between HCY and PIH. Interestingly, Maru et al.'s [14] study found that elevated HCY levels were associated with a range of maternal complications, including abruption, retinopathy, MODS, maternal mortality, and eclampsia. Our study did not examine the relationship between HCY and these specific complications, but our results suggest that HCY may be a useful marker for identifying patients at risk of HDCP. 

Hosseini et al.'s study [15] found that low plasma FIB levels were associated with postpartum haemorrhage (PPH) and severe postpartum haemorrhage (sPPH) in a cohort of 169 term pregnant women undergoing elective Cesarean section. Interestingly, our study found that serum FIB levels were positively correlated with HDCP (r = 0.547, P<0.05), whereas Hosseini et al.'s study found that low plasma FIB levels were associated with PPH and sPPH. This suggests that FIB may play a different role in the pathophysiology of HDCP and PPH. However, both studies highlight the importance of FIB as a biomarker for predicting adverse pregnancy outcomes.

This work found that serum lipid indicators, including TG, TC, HDL, and LDL levels in HDCP patients, were greatly higher versus normal pregnancies, and they all serve as RFs for HDCP. Wei et al. [16] also observed significantly elevated pre-pregnancy serum TC, TG, and LDL-C in HDCP patients, with early lipid levels predicting HDCP in twin pregnancies. This suggests that HDCP is often associated with lipid metabolism disorders.

Disruption of the body's hemostatic system can lead to a hypercoagulable state, an independent RF for developing HDCP [17]. Following the onset of HDCP, patients experience spasmodic constriction of systemic arterioles, leading to endothelial cell damage due to hypoxia and increased blood viscosity. FIB adheres to the matrix during this process and becomes depleted [18]. HDCP patients exhibit significantly increased APTT and PT, with decreased consumptive FIB, indicating a coagulopathic state that increases the risk of disseminated intravascular coagulation (DIC) [19]. In DIC, fibrin clots block microvessels and cause organ damage.

Untimely diagnosis and treatment of DIC can result in maternal mortality [20] [21]. Clinically, assessments often include the coagulation parameters PT, APTT, thrombin time (TT), and FIB to evaluate pregnancy complications. As gestational age increases, maternal PT, APTT, and TT gradually shorten while FIB levels rise, indicating an intensification of maternal hypercoagulability with advancing gestation, which then resolves postpartum as coagulation parameters return to normal [22]. This work observed that HDCP patients exhibit considerably shorter APTT and PT compared to normal pregnancies, along with dramatically elevated FIB levels. This indicates a heightened hypercoagulable state in HDCP patients, emphasizing the need for timely treatment and prevention of DIC. FIB is also an inflammatory response protein, and its rapid increase in serum levels may be an RF for atherosclerosis [23]. HDCP patients experience endothelial damage, leading to inflammation and monocyte aggregation, resulting in elevated FIB levels due to the release of inflammatory mediators [24]. This work found a positive correlation between FIB and HDCP, highlighting FIB as an RF influencing HDCP. This association may be attributed to serum FIB binding specifically with immune cells such as neutrophils, promoting leukocyte adherence to endothelial cells, thereby causing vascular narrowing and elevated blood pressure [25].

β-HCG is secreted by syncytiotrophoblast cells, entering the maternal circulation through the placental intervillous space and serving as an important indicator of placental function. In HDCP, systemic arteriolar spasm significantly reduces uteroplacental blood flow, leading to focal degeneration and necrosis of the placental villi. This, in turn, triggers the rapid proliferation of syncytiotrophoblast cells and their transformation into syncytial knots, inducing the release of β-HCG [26] [27] [28]. This work found that serum β-HCG levels in HDCP patients were markedly higher versus normal pregnancies, with a positive correlation between b-HCG and HDCP, thus identifying β-HCG as an RF influencing HDCP. Wang et al. [29] further noted a correlation between elevated β-HCG levels and the severity of HDCP, while peripartum β-HCG levels correlated with inflammation and oxidative stress markers. Huang et al. [30], through a metaanalysis, systematically evaluated pregnancy outcomes in women with high β-HCG levels and found significantly higher rates of HDCP, preterm birth, miscarriage, gestational diabetes, and fetal growth restriction compared to women with low β-HCG levels. Uteroplacental ischemia-hypoxia can easily trigger the development of HDCP, where hypoxia in trophoblast cells promotes increased proliferation to maintain normal placental function, resulting in a sharp rise in β-HCG levels [31]. This indicates an association between β-HCG levels and pregnancy complications and adverse outcomes, highlighting the importance of close monitoring and timely intervention for women with high β-HCG levels.

HCY is an intermediate product of methionine metabolism in the body, primarily metabolized by the kidneys. Elevated HCY levels readily undergo oxidation reactions, producing hydrogen peroxide, superoxide radicals, and other by-products, leading to endothelial damage and dysfunction. This disrupts the balance of vascular active substances, inhibits vasodilation, and promotes hypertension [32]. HCY increases platelet adhesion, leading to the formation of atherosclerotic plaques or thrombosis [33]. This work found that serum HCY in HDCP patients was dramatically superior to normal pregnancies, and there was a positive correlation between HCY and HDCP, identifying HCY as an RF influencing HDCP. Zeng et al. [34] found that HCY levels in HDCP patients were markedly superior to normal pregnancies, and serum HCY levels correlated positively with disease severity. HCY can inhibit nitric oxide (NO) release, leading to impaired vasodilation, and can also stimulate excessive proliferation of vascular smooth muscle cells, resulting in thickening and narrowing of blood vessel walls, ultimately causing elevated blood pressure [35]. Sayyah-Melli et al. [36] observed that supplementation with folic acid during pregnancy can lower concentrations of HCY at delivery and lower the incidence of adverse pregnancy outcomes such as early miscarriage. Hence, the relationship between folic acid supplementation and HDCP needs further exploration.

## Conclusion

In conclusion, pregnant women with HDCP exhibit significant lipid metabolism dysregulation and elevated FIB, β-HCG, and HCY serum levels. Serum levels of FIB, β-HCG, and HCY are associated with HDCP and serve as independent RFs influencing HDCP. However, this study has certain limitations, necessitating the inclusion of additional data to analyze the relationship between these indicators and adverse pregnancy outcomes in HDCP patients. This research contributes to identifying serological markers for early screening and prediction of HDCP.

## Dodatak

### Conflict of interest statement

All the authors declare that they have no conflict of interest in this work.

### List of abbreviations

APTT, Activated Partial Thromboplastin Time;<br>β-HCG, beta-human chorionic gonadotropin;<br>BMI, Body Mass Index;<br>CG, Control Group;<br>DIC, Disseminated Intravascular Coagulation;<br>EDTA, Ethylenediaminetetraacetic acid dipotassium salt;<br>FIB, Fibrinogen;<br>HCY, Homocysteine;<br>HDCP, Hypertensive Disorder Complicating Pregnancy;<br>HDL, High-Density Lipoprotein;<br>LDL, Low-Density Lipoprotein;<br>NO, Nitric Oxide;<br>OR, Odds Ratio;<br>PT, Prothrombin Time;<br>RF, Risk Factor;<br>ROC, Receiver Operating Characteristic;<br>SPSS, Statistical Package for the Social Sciences;<br>TC, Total Cholesterol;<br>TG, Triglyceride;<br>TT, Thrombin Time.
